# The Impact of P-Glycoprotein on Opioid Analgesics: What’s the Real Meaning in Pain Management and Palliative Care?

**DOI:** 10.3390/ijms232214125

**Published:** 2022-11-16

**Authors:** Flaminia Coluzzi, Maria Sole Scerpa, Monica Rocco, Diego Fornasari

**Affiliations:** 1 Department Medical and Surgical Sciences and Biotechnologies, Sapienza University of Rome, Polo Pontino, 04100 Latina, Italy; 2 Unit of Anaesthesia, Intensive Care, and Pain Medicine, Sant’Andrea University Hospital, 00189 Rome, Italy; 3 Department of Surgical and Medical Science and Translational Medicine, Sapienza University of Rome, 00189 Rome, Italy; 4 Department of Medical Biotechnology and Translational Medicine, Università degli Studi di Milano, 20129 Milan, Italy

**Keywords:** P-glycoprotein, opioids, chronic pain, drug-drug interactions, PAMORAs, neuropathic pain, blood brain barrier, ATP-binding cassettes, polymorphisms, inflammation

## Abstract

Opioids are widely used in cancer and non-cancer pain management. However, many transporters at the blood–brain barrier (BBB), such as P-glycoprotein (P-gp, ABCB1/MDR1), may impair their delivery to the brain, thus leading to opioid tolerance. Nonetheless, opioids may regulate P-gp expression, thus altering the transport of other compounds, namely chemotherapeutic agents, resulting in pharmacoresistance. Other kinds of painkillers (e.g., acetaminophen, dexamethasone) and adjuvant drugs used for neuropathic pain may act as P-gp substrates and modulate its expression, thus making pain management challenging. Inflammatory conditions are also believed to upregulate P-gp. The role of P-gp in drug–drug interactions is currently under investigation, since many P-gp substrates may also act as substrates for the cytochrome P450 enzymes, which metabolize a wide range of xenobiotics and endobiotics. Genetic variability of the ABCB1/MDR1 gene may be accountable for inter-individual variation in opioid-induced analgesia. P-gp also plays a role in the management of opioid-induced adverse effects, such as constipation. Peripherally acting mu-opioid receptors antagonists (PAMORAs), such as naloxegol and naldemedine, are substrates of P-gp, which prevent their penetration in the central nervous system. In our review, we explore the interactions between P-gp and opioidergic drugs, with their implications in clinical practice.

## 1. Introduction

Opioids still represent the cornerstone of chronic pain management; however, as pain is a multidimensional experience, different factors may make pain management difficult, particularly in patients with cancer [1]. Drug–drug interactions represent one of the main difficulties in optimizing analgesia in patients with comorbidities and under polypharmacy, therefore accurate knowledge of the pharmacokinetics of analgesics is a key point for therapy personalization [2]. In the last few years, the importance of the interaction between mu-opioid receptor (MOR) agonists and P-glycoprotein (P-gp), as an efflux protein limiting access through the blood–brain barrier (BBB), has been an interesting topic of research in terms of analgesic activity and innovative strategies for managing opioid-related side effects. Many central nervous system (CNS) drugs, currently used by cancer pain patients, have some affinity for P-gp, such as certain anticancer drugs, antidepressants, and HIV-protease inhibitors. This review focuses on pre-clinical and clinical evidence on the role of P-gp substrate activity of clinically relevant MOR agonists and antagonists.

## 2. What Is P-gp?

The BBB works as an interface between blood and the brain, protecting the CNS from pathogens, toxins, injury, and diseases [3]. The non-fenestrated endothelium of the BBB is made of transmembrane proteins [4], creating tight junctions and thus restricting paracellular movement [5]. Hence, most compounds reach the brain via the trans-cellular route, while only lipophilic molecules can cross the endothelium via mere passive diffusion [6]. Moreover, endothelial cells are interconnected by adherens junctions and surrounded by pericytes, astrocytes, microglia, neurons and the extracellular matrix, with all these elements contributing to creating the so-called “neurovascular unit” (NVU) [4,5]. Among all transporters distributed along the BBB, ATP-binding cassettes (ABCs) are widely expressed on endothelial cells and in the luminal plasma membrane of brain parenchyma, and are responsible for transporting many different compounds [3]. P-gp is the most expressed ABC transporter in the BBB, both in humans and in mice [7].

P-gp, the encoded product of the multidrug-resistance gene 1 (MDR1), is a 170 kDa [8] N-glycosylated membrane protein, made of 1280 amino acids, with two halves connected by a flexible linker and with a 65% sequence homology. Each half contains a transmembrane domain (TMD) and a nucleotide-binding domain (NBD), the latter lying on the cytosolic side of the cell membrane [9,10]. The two TMDs form a 6000 Å cavity, big enough for P-gp to bind to numerous substrates concomitantly [9]. Each TMD contains six transmembrane helices [8,11], which are connected by extracellular loops (ELs) in the periplasm and intracellular helices (IHs), also called intracellular loops (ICLs), in the cytoplasm [12]. Each of the two NBDs is made of a highly-conserved sequence, comprising the Walker A and Walker B motifs, the A-, Q-, and H-loops for one NBD, and the D-loop and signature consensus sequence (C motif) LSGGQ for the other NBD [12,13]. According to the “ATP-switch model”, the two NBDs bind to ATP via rotational and translational movements, and consequently dimerize, resulting in an “ATP-sandwich structure”, while the TMDs turn outwards via reorientation of the TM helices, in order to expose and unidirectionally extrude the substrate to the extracellular compartment. After that, ATP hydrolysis forces the NBDs to dissociate, so that the TMDs can flip inwards again [12]. Based on the spatial conformation of the NBDs, three general conformations of P-gp are possible: in the “closed” conformation, the NBDs bind and hydrolyze ATP, and the cavity is exposed to the extracellular space, where substrates are accessible; conversely, the cavity turns to the cytosol and the NBDs separate in the “open” conformation, when ligands are not present or act as P-gp inhibitors. When ATP is available but there are no ligands, P-gp is in an intermediate conformation between the outward- and the inward-facing states [9]. P-gp uses a twist-and-squeeze mechanism to export hydrophobic drugs out of cells. When a hydrophobic substrate enters the inner cavity, through the gate open to the inner leaflet of the membrane, it is attracted at the top of the cavity, where it binds to the aromatic hydrophobic networks and triggers the conformational change from the inward-open to the outward-open state [14]. P-gp works as a lipid flippase, shuttling cholesterol and phospholipids from the inner to the outer leaflet of the cell membrane [15].

Besides being expressed on the plasma membrane (PM) of different cell types, P-gp is also detectable in intracellular compartments, namely the nucleus, the endoplasmic reticulum (ER) and the Golgi, where the protein itself is first synthesized and then modified. P-gp is synthesized as a coreglycosylated compound with a 150 kDa molecular weight and is associated with chaperons such as heat shock cognate 71 kDa protein (Hsc70) and calnexin [10]. Successive transfer outside the nucleus may be cargo or Hsc70-dependent [16]. After being folded in the ER, the intermediate protein is additionally glycosylated in the Golgi, thus resulting in the final 170 kDa form [10]. Glycosylation seems to be necessary for P-gp to be transported to the membrane, rather than for its efflux activity [5]; nonetheless, glycosylated P-gp is able to extrude chemotherapeutic drugs when located on the nucleus membrane, thus resulting in resistance to chemotherapy [16]. Intracellular trafficking between different compartments and recycling of P-gp occurs through endosomes and vesicles and was found to be Early Endosome Antigen 1 (EEA1) or Ras-associated binding protein (Rab)-mediated in different cell types [10,16]. Whether P-gp is expressed in mitochondria has been quite a controversial matter: P-gp may pump compounds out of mitochondria as a protective system or into them in order to sequester toxic molecules. While some authors claim that detection of P-gp in these organelles derives from plasma membrane contamination [17], other studies have assessed its presence, especially after exposure to oxidative stress [18], and its activity has been associated with chemotherapy resistance in tumor cell models [19]. Furthermore, P-gp is detectable in lysosomes, where it gets degraded. P-gp may also be eliminated through ubiquitination and successive removal in proteasomes, especially if it leaves the ER in a misfolded or non-glycosylated form. However, exposure to P-gp substrates and its phosphorylation by kinases may preserve it from being degraded [5,10]: in fact, mature P-gp on the plasma membrane (PM) is phosphorylated on the linker between its two halves [5].

As an ABC, P-gp is able to transport many different molecules against their concentration gradient via ATP hydrolysis from the BBB endothelium back to the systemic circulation [8,20]. This unidirectional transport [21] limits the cell uptake, distribution, bioavailability [22] and accumulation of many compounds within the brain, including potentially toxic xenobiotics. In cancer cells, however, P-gp expressed in the lysosomal membrane may sequester chemotherapeutics, resulting in drug resistance. This P-gp-mediated lysosomal drug trapping has been recognized as a second-line defence in brain capillary endothelial cells. These complexes of substrate/lysosomal P-gp are exposed at the apical side of the cell membrane and undergo phagocytosis by neutrophils [23].

There are no data about P-gp being expressed in brain cells other than the BBB endothelium. Nonetheless, it has been found to co-localize with glial fibrillary acidic protein, which is expressed in astrocytes [8], and it may be present on pericyte processes reaching endothelial cells [16]. Other than in the BBB, P-gp is expressed in the lungs [24], adrenal glands [25], placenta and fetal membranes [26], as well as other organs with excretory functions, such as bile and pancreatic ductules, and kidney proximal tubules, thus allowing the secretion of metabolites and xenobiotics into the bile and urine, respectively [8,22]. In the intestine, P-gp is distributed according to an expression gradient: negligible or very low expression in the first part of small intestine (duodenum and proximal jejunum) and high expression in the distal tract of ileum and colon. In the small intestine, P-gp is mainly located in the brush border surface of enterocytes where it acts as a barrier against exogenous compounds. This implies that the impact of P-gp on oral bioavailability may also depend on the site of drug absorption [27,28]. P-gp may limit the bioavailability of many substrates, by pumping them out of the enterocytes into the lumen, thereby prolonging their exposure to CYP3A4 [29]. Since transport activity of P-gp in the intestinal lumen becomes saturated by high drug concentrations, its impact is minimum for drugs with rapid absorption and requiring high dosages. On the other hand, P-gp considerably hampers absorption of drugs requiring low doses or with slow diffusion rates [22]. Recent efforts have focused on developing lipid-based formulations for oral drugs, such as liposomes, complexes, water-soluble prodrugs, and salts, designed to rapidly dissolve in the stomach and maintain a supersaturated state in the duodenum for an extended time, thus increasing lymphatic absorption and/or passive diffusion [30].

An approximate 10-fold inter-individual variability exists in intestinal P-gp expression. Different disease states are associated with altered intestinal P-gp expression and function, such as hepatic and renal failure, diarrhea, colorectal carcinoma, inflammatory bowel disease (IBD), organ transplants, ischemia/reperfusion, and obesity. Pathological conditions and endogenous inhibitors, such as uremic toxins and bilirubin, may hamper P-gp function, and thus the latter does not always correlate with mRNA levels. P-gp function displays a daily rhythm, which is not affected by sleep, but is probably influenced by neurotransmitters, cytokines, and hormones [31]. Drug daily dosing time and formulations may alter P-gp expression: for instance, drugs taken in the evening may have lower bioavailability. Thus, personalized therapies are warranted [32].

The mechanism of action of P-gp is still not perfectly established: two models have been proposed. The first one refers to P-gp as an “hydrophobic vacuum cleaner”, carrying its substrates from the lipid bilayer to the external aqueous medium. Conversely, according to the “flippase model”, P-gp is able to flip its substrates from the inner to the outer leaflet of the lipid bilayer of the plasma membrane or into the extracellular milieu [8,33].

Both endogenous (namely steroid hormones such as corticosteroids, bilirubin, cytokines, peptides) and exogenous (e.g., drugs, such as vinca alkaloids, anthracyclines, digoxins, opioids and so on) compounds are P-gp substrates. P-gp substrates are generally amphipatic [5,7,8,22], positively charged at physiological pH [8] and with a molecular weight ranging from 250 to 1202 g/mol [22]. Aromatic and hydrophobic interactions are crucial for substrate binding to P-gp, with van der Waals and hydrophobic residues such as phenylalanine being the main contributors [34]. P-gp substrates generally have an efflux ratio higher than 1.5, this value decreasing to 1 when P-gp is inhibited, and their distribution is enhanced in P-gp deficient/knockout animals; they usually upregulate P-gp when administrated continuously [33,35].

### 2.1. P-gp Regulation

P-gp may be upregulated in certain pathological conditions, and/or as a consequence of prolonged exposure to xenobiotics, including drugs. Rifampin is recognized as the most potent P-gp inducer, with an approximate 20–67% reduction in P-gp substrate exposure, while other inducers cause a 12 to 42% reduction [36].

Multidrug resistance is still one the main problems for cancer treatment, since many chemotherapeutic agents are P-gp substrates and P-gp is overexpressed in cancer cells, hampering the internalization and leading to resistance to chemotherapeutic drugs; hence, recent research has focused on developing P-gp inhibitors [37].

P-gp is also upregulated in several neurological disorders, such as Parkinson’s disease [38], Alzheimer’s disease [39], amyotrophic lateral sclerosis (ALS) [40], and epilepsy. Many antiepileptic drugs (AEDs) are P-gp substrates, and their repeated administration leads to P-gp upregulation, thus resulting in drug resistance [41]. Recent clinical trials suggest the use of cannabinoids in refractory epilepsy, although the possible mechanism of action is still unclear [42]. In cultured vascular endothelial cells, cannabidiol has been shown to inhibit the efflux of the P-gp dependent rhodamine-123, similarly to the P-gp inhibitor tariquidar [43].

Treatment of infectious conditions may fail since antimicrobials are P-gp substrates: coadministration with P-gp inhibitors may be a solution to reduce the minimum inhibitory concentrations (MICs) of antimicrobials and increase the susceptibility of microorganisms towards antimicrobial drugs [22]. P-gp overexpression may also complicate antiretroviral therapies (ARTs), since many antiretrovirals (ARVs), such as protease inhibitors (PIs), non-nucleoside reverse transcriptase inhibitors (NNRTIs), and nucleoside/nucleotide reverse transcriptase inhibitors (NRTIs/NtRTIs), are P-gp substrates and may also enhance its expression [44,45]. P-gp regulation by PIs may be cell-type-dependent and “biphasic”: ritonavir caused an initial 2.8-fold increase in fexofenadine (a P-gp substrate) area under the curve (AUC) in vivo, while this became lower after 2 weeks in the steady state, stabilizing at a 1.4-fold increase. These results are unlikely due to effects on CYP3A4 and renal P-gp, while they are more probably due to P-gp affecting enterocytes and the canalicular side of hepatocytes. Similar results were obtained with verapamil and lopinavir/ritonavir towards fexofenadine AUC and are attributed to a mild P-gp induction in the steady state [46]. When co-administrated, PIs may have different effects on P-gp, either inhibiting or increasing it [45]. Since HIV is among the infectious complications of intravenous drugs use (IDU), and with the rise of the “opioid epidemic”, ARVs may often be administrated alongside methadone for OUD maintenance therapy [47], either leading to opioid withdrawal and/or a change in drug plasma concentrations [45].

Similarly, when opioids are administrated for chronic pain management, P-gp upregulation was found to play a role in opioid tolerance development [3].

### 2.2. P-gp Inhibition

Inhibiting or “by-passing” P-gp may be a valid therapeutic strategy in many clinical conditions [48]. However, use of known P-gp inhibitors is associated with high risk of infection and systemic toxicity, with possible fatal outcomes [4,49]. Nowadays, no P-gp inhibitors have been approved for clinical use [50].

Inhibiting P-gp may significantly increase the amount of drugs available in the CNS. Mdr1a (−/−) knockout mice have been shown to have higher brain levels of several drugs (digoxin, vinblastine, cyclosporine A) [51], supporting the hypothesis that the transport of P-gp substrates to the brain is enhanced in case of P-gp deficiency/absence [7].

P-gp can be inhibited by blocking the drug binding site either competitively, non-competitively, or allosterically [22], as well as by interfering with ATP hydrolysis and ATP-dependent thermostabilization of P-gp [52]. Since most P-gp inhibitors are also P-gp substrates, specific compounds should be created with large molecular differences from therapeutic drugs, so that P-gp shall be able to discriminate between these two compounds.

P-gp inhibitors can be classified into four generations, based on their selectivity, potency, and drug–drug interaction (DDI) potential [50].

First generation inhibitors include cyclosporine A, quinidine, verapamil (VRP), and tamoxifen. Since their affinity for P-gp is low, they only inhibit P-gp when administrated at doses much higher than the therapeutic ones, with consequent high risk for toxicity and DDIs. Second-generation compounds, namely PSC833 (valspodar) and (R)-verapamil, have been derived from structural modifications of first-generation inhibitors, and showed greater affinity to P-gp; still, pharmacokinetic interactions via cytochrome P450 enzymes and/or ABC transporters were detected. Third-generation inhibitors with high affinity and potency have been developed, including laniquidar (R101933), tariquidar (XR9576), zosuquidar (LY335979), encequidar (HM30181), and elacridar (GF120918) [22,50]. However, these compounds display toxicity and scant clinical benefits. Finally, fourth-generation inhibitors have been developed, including: (1) peptidomimetics; (2) molecules isolated from natural sources, and their derivatives (i.e., polyphenols, coumarins, terpenoids); (3) “dual” ligands, which are able to inhibit both P-gp and other targets, such as tyrosine kinase inhibitors (TKIs) [50,53]. Other molecules, namely A3 adenosine receptor agonists, have been developed as treatment options for specific conditions, e.g., cancer, chronic pain, and immune disorders, and they coincidently also inhibit P-gp [13]. Ideal P-gp inhibitors shall be able to selectively counteract it in pathological circumstances, while maintaining its basal activity under normal conditions, in order to eject pathogens and toxic compound out of the CNS [16]. Recent literature has focused on the role of nanocarriers [54,55] and small interfering RNA (siRNA) [56,57] in P-gp inhibition. Moreover, inducing structural changes in the lipid composition of plasma membranes, they may impact P-gp conformation and activity [58,59]. Monoclonal antibodies against P-gp/MDR1, namely MRK-16, MRK-17, IUC2, 4E3 [22,60], and conjugated antibodies [61,62], have been investigated to overcome drug resistance. Modulating P-gp synthesis and regulatory pathways or keeping P-gp bound to caveolar proteins may avoid monomers becoming liberated and active. Forcing intracellular accumulation of inactive P-gp by inhibiting its maturation could be an alternative [10,63,64].

### 2.3. P-gp and Pain/Inflammation

Λ-Carrageenan (CG)-induced hindpaw inflammation is a useful rat model of localized inflammatory pain and thermal hyperalgesia, which can be used to elucidate the impact of peripheral inflammatory pain (PIP) on P-gp expression and activity [64], mainly via post-transcriptional phenomena [16].

Normally, P-gp is located in caveolae throughout the PM of endothelial cells, alongside caveolin1 (CAV-1), polymerase 1 and transcript release factor (PTRF/cavin1), serum deprivation response protein (SDPR/cavin2), and protein kinase C delta binding protein (PRKCDBP/cavin3), all contributing to the regulation of signaling pathways and protein trafficking [63]. The N-terminal portion of P-gp contains CAV1-binding motif. When CAV-1, which is a key trafficking protein, binds to P-gp, this is negatively regulated, as CAV-1 facilitates the sequestration of P-gp as a high-molecular-weight (>250 kDa), disulfide-bonded complex. The interaction between P-gp and CAV-1 is enhanced by tyrosine-14-phosphorylation of CAV-1 and negatively regulates P-gp [65].

Inflammatory conditions and PIP-induced CAV1 phosphorylation on tyrosine-14 and a reduction in disulphide-bond complexes, with consequent dynamic redistribution of P-gp between subcellular compartments, especially from the nucleus towards lower density and cholesterol-rich membrane sections, lead to a nearly 40% P-gp activity enhancement [63,64]. In summary, peripheral inflammatory hyperalgesia promotes significant changes in the P-glycoprotein trafficking at the BBB, leading to a pathological increase in the P-gp expression, which could affect the efficacy of drugs that need to enter the CNS to perform their therapeutic effect.

Not only do inflammatory acute conditions upregulate P-gp, but they also induce other extensive changes in the structure of the BBB, for instance by altering expression of occludins, claudins, and zonula occludens-1 (ZO-1) [3,66]. Whether inflammatory acute pain also affects the integrity of the blood–spinal cord barrier (BSCB) is still unclear. Interestingly, perineural administration of local anesthetics, such as bupivacaine 0.75%, in the lambda-carrageenan PIP model, was shown to prevent the aforementioned structural changes in the BBB and to significantly reduce thermal allodynia [67]. These observations suggest a possible role of nociceptive signaling for the alteration of BBB under inflammatory conditions.

The non-steroidal anti-inflammatory drug (NSAID) diclofenac was associated with P-gp overexpression at the BBB in a λ-carrageenan-induced PIP rat model, thus hampering morphine intake to the brain, when this opioidergic drug was administrated 3 h after diclofenac exposure. Conversely, morphine-induced antinociception was not hindered when both drugs were administrated simultaneously. Similar findings were detected in Caco-2 human intestinal cells. Diclofenac may regulate P-gp through at least two mechanisms. Diclofenac is a well-known COX-2 enzyme inhibitor, thus it reduces prostaglandins levels and shifts towards lipoxygenase-mediated production of leukotrienes, which are able to promote P-gp expression after binding to their nuclear receptors [68]. Moreover, diclofenac may induce overexpression of TNF-α, which, by binding to TNF receptor 1 (TNF-R1), increases the production of endothelin-1 (ET-1). ET-1, by binding to ETB receptor, activates protein kinase C (PKC) and nitric-oxide synthase (NOS) enzymes and eventually increases the transcription nuclear factor NF-κB, which was found to act on the BBB as a protector against brain damage (e.g., hypoxia, stroke) and toxicity [69] and may promote MDR1 transcription through the PGE2-EP1-PI3K/Akt pathway [70]. The activation of this signaling cascade was associated with higher P-gp expression 6 h after exposure to TNF-α or ET-1; conversely, when different steps of this pathway were blocked or inhibited, Pgp upregulation was avoided or reverted [69].

The activation of other signaling pathways, namely the glucocorticoid receptor (GR), constitutive androstane receptor (CAR), aryl hydrocarbon receptor (AhR), and pregnane R receptor (PXR), leads to P-gp upregulation via enhanced nuclear transcription of the MDR1 gene [3,7,71]. Administration of different substrates of these receptors, including both natural compounds and drugs [72,73], led to increased P-gp activity in a time- and dose-dependent, reversible manner in animal models [20,74].

Moreover, MDR1 mRNA and P-gp protein expression were found to be enhanced after 1α,25-dihydroxyvitamin D3 [1,25(OH)2D3]-mediated activation of the vitamin D receptor (VDR) [75]. Osteoporosis is widely recognized as a cause of chronic skeletal pain, especially when bone fractures occur, often resulting in the need for strong analgesic prescription [76], presumably alongside vitamin D replacement. If vitamin D does upregulate P-gp, then tolerance to opioids may occur, potentially forcing physicians to increase opioid dosage. Several in vitro and in vivo studies in the last years have pointed out that opioids themselves may hamper bone formation and healing with a dose-dependent pattern [77]. Even though data on this matter are still quite controversial, maintaining opioid doses as low as possible throughout the duration of the therapy is unanimously acknowledged as the best way to avoid opioid-related adverse effects [78].

### 2.4. P-gp Polymorphisms

Genetic variants of the MDR1 gene may alter the expression and/or activity of P-gp [3], thus interfering with the absorption, distribution and excretion of P-gp substrates [8]. Thirty-eight single nucleotide polymorphisms (SNPs) on the coding region of ABCB1 have been reported: the C3435T on exon 26 (rs1045642) [11,79], G2677T (rs2032582) on exon 21 [11,45], and C1236T (rs1128503) on exon 12 [11] are the most common and most studied variants until now, with diverse allelic frequencies in different populations [11,79]. Low-haplotype diversity was observed in Caucasians. A haplotype containing the subset 1236T-2677T-3435T is highly represented among non-African populations, while the sub-haplotype 1236C-2677G-3435C is common in African-American populations. The three most common SNPs in the ABCB1 gene are well represented both in Caucasians and Ashkenazi Jewish. Frequency of the 3435T allele in Ashkenazi, Yemenite, North American and Mediterranean Jewish populations is similar; the 3435T allele is more frequent in near Eastern Jews [11]. The 3435G allele is associated with higher P-gp mRNA levels in human enterocytes, while 3435TT carriers have a 2-fold reduced expression of duodenal P-gp [11,45]. A haplotype with the promoter SNP 129T > C (rs3213619) was correlated with high levels of P-gp expression and its increased activity, independent of 3435C > T. Variants 1236T, 2677T and 3435T lowered P-gp activity in vitro in a substrate-specific manner [11]. P-gp co-localizes with CYP3A4 in enterocytes, and both were found to be induced after xenobiotic exposure. Moreover, MDR1 2677TT carriers had higher CYP3A4 expression [45]. P-gp and CYP3A4 share common substrates, and are both responsible for their distribution, metabolism, and elimination. Their polymorphisms were associated with higher risk of chemotherapy-induced peripheral neuropathy (CIPN) in taxane-treated patients. Particularly, ABCB1 3435 and 1236 TT genotypes were associated with reduced P-gp expression and consequent higher taxane plasma levels and taxane-related adverse effects, such as diarrhea and neutropenia [80,81].

Allelic variants of P-gp are also associated with altered P-gp expression at the BBB, thus affecting drug delivery to the CNS [7], as is the case for opioids, hence resulting in inter-individual variability in pain relief [3]. 1236TT, 2677TT, and 3435TT carriers (also referred to as “TT-TT-TT” haplotype) need higher methadone doses to avoid withdrawal [11], probably associated with faster metabolism and consequent lower methadone plasma levels [79]; conversely, heterozygous subjects for these three SNPs have an approximately 3-fold possibility of stabilizing at lower methadone dosage. SNP 1236C > T is a synonymous variant, located in one of the intracellular loops of the protein, next to an ATP-binding/utilization domain. 1236C > T may not change the protein sequence, but it may affect P-gp translation regulation and RNA stability [11]. On the other hand, homozygous C3435T TT carriers also had better analgesic effects with morphine administration, compared to wild-type CC subjects [82]; however, they were found to have higher risk of persistent postoperative pain [83]. These apparently controversial findings derive from studies conducted on subjects of different races.

### 2.5. Opioids and P-gp

Opioids are the stronger analgesic drugs available for treating moderate to severe acute and chronic pain. They work as analgesics, by potentiating the physiological endogenous modulating system through their interaction mainly with the mu-opioid receptor (MOR). Opioid receptors are located on the primary afferent fibers (PAF), where they prevent calcium influx in the first-order neuron and the release of excitatory neurotransmitters, such as glutamate and substance P. Opioid receptors are also expressed by second-order neurons, in the dorsal horn, where they activate G protein gated inwardly rectifying potassium (GIRK) channels, which hyperpolarize neurons [84]. Their analgesic reward effects are dependent on the rate and speed of drug crossing the BBB and accessing the CNS. Lipophilicity of the different compounds contributes to differences in the speed at which opioids can cross the BBB. Similarly, P-gp may play a central role in opioid analgesia, according to its ability to extrude the drug from the CNS [85].

**Figure 1:**
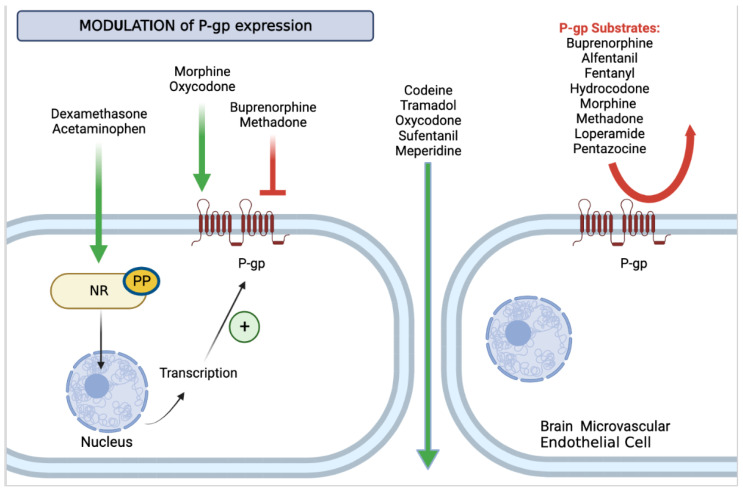
Modulation of P-gp expression at the BBB by analgesics. P-gp limits opioid distribution in the brain. (**Right Side**) Most opioids are known to be P-gp substrates (red arrow), therefore, increased expression of P-gp potentiates their efflux and prevents their delivery in the central nervous system. Other opioids are not P-gp substrates (green arrow) and this could explain the faster onset of action. (**Left Side**) Chronic opioid exposure may also modulate P-gp activity: morphine and oxycodone are P-gp inducers (green arrow), while buprenorphine and methadone are inhibitors (red inhibition arc) of ATP-Binding Cassette (ABC) trasporters at the blood-brain barrier (BBB). Other drugs commonly used in pain management may modulate P-gp expression at the BBB, through intracellular mechanisms of nuclear transcription. Dexamethasone and paracetamol bind the nuclear receptor (NR), which, after dephosphorylation by a protein phosphatase (PP), translocates to the nucleus and binds its response element on a target gene, leading to protein transcription and overexpression of ABC transporters, such as P-glycoprotein (P-gp) on the membrane of brain microvascular endothelial cells.

P-gp limits opioid distribution in the brain (Figure 1). On the other hand, both endogenous and exogenous opioidergic compounds were found to regulate P-gp activity. Among endocannabinoids, anandamide (AEA) inhibited P-gp in HK-2-immortalized cells, which have similar characteristics to in vivo proximal tubules. Conversely, 2-arachidonoylglycerol(2-AG) and palmitoylethanolamide (PEA) did not inhibit P-gp [86].

Chronic exposure to opioids may result in P-gp overexpression in different areas of the brain, especially the cortex, hippocampus and blood vessels [48], and consequent extrusion of opioids themselves outside of the CNS, leading to opioid tolerance, with higher doses needed to achieve analgesia [7]. Opioid administration is associated with P-gp overexpression via activation of several signaling pathways. For instance, opioids activate toll-like receptors (TLRs), especially TLR4 and its coreceptor myeloid differentiation factor-2 (MDF-2). Consequent astrocyte and microglia activation leads to secretion of inflammatory cytokines, which upregulate P-gp in a species- and time-dependent manner. Eventually, these events are associated with opioid tolerance and withdrawal. On the contrary, high doses (100 mg/Kg) of naloxone were found to inhibit TLR4, thus attenuating allodynia and hyperalgesia, and enhancing opioid-derived analgesia [3,87]. Naloxone-precipitated morphine withdrawal in animal models was the result of a NMDA-mediated increase in extracellular glutamate concentrations in several brain areas, such as the locus coeruleus, hippocampus and nucleus accumbens [3,7,82]. Such glutamate increase led to COX-2 signalling activation and, eventually, P-gp overexpression, the latter being reversible after administration of NMDA or COX-2 antagonists and inhibitors [3,7,70].

Most studies regarding the relationship between P-gp and opioids revolve around morphine, which acts as a P-gp substrate both in vitro [88] and in vivo [89].

P-gp knockout (KO) mice displayed higher brain levels of morphine, compared to wild-type mice, thus resulting in enhanced morphine-induced analgesia [33], the latter being negatively correlated with cortical P-gp expression and activity [89]. Accordingly, P-gp inhibition resulted in increased transport rates of morphine through the BBB, thus enhancing its analgesic and acute locomotor effects [21], and preventing tolerance [33]. On the other hand, P-gp inhibition was associated with morphine-induced reinforcement processes in the nucleus accumbens and the dorsal striatum in mice models [21].

Intestinal P-gp impedes oral morphine absorption, reducing its bioavailability [7]: this effect can be reverted by coadministration of a P-gp inhibitor, which results in increased morphine plasma concentrations [3,33] and antinociceptive effects [83].

Morphine does not act as a P-gp inhibitor [7]. Its administration leads to increased mRNA and protein P-gp levels [3], as well as enhanced PIP-mediated transport of P-gp reservoirs from the nucleus to the PM [48]: consequent P-gp overexpression [33,45] and enhanced ATPase activity results in reduced morphine-induced analgesia. Opioid suspension brings P-gp back to normal levels [89]. Morphine-induced P-gp upregulation may be cell-type- and time-dependent. A 5-day treatment with morphine upregulated P-gp mRNA levels by 1.2-fold, in rat brain microvessels, even after treatment discontinuation. This suggests that P-gp upregulation may be correlated with morphine withdrawal, rather than cause tolerance development [3,87].

Data regarding active morphine metabolite morphine-6-glucuronide (M6G) are still controversial since it was not found to be a P-gp substrate in mice and humans. On the other hand, P-gp inhibition increased its spinal cord levels in rats, and it seems to act as a P-gp inhibitor in vitro [3]. M6G could still be a substrate for other efflux transporters at the BBB [4].

Conversely to morphine, codeine is not a P-gp substrate, since verapamil does not alter its transport in Caco-2 cells and immortalized rat brain endothelial cells (RBE4) [7]. Codeine stimulates P-gp mediated hydrolysis, but it is not actively effluxed by P-gp; therefore, it should be considered a nontransported P-gp substrate [90], and this feature could explain the faster onset of action compared to an equianalgesic dose of morphine [91].

Whether oxycodone is a P-gp substrate is still unclear, since different P-gp inhibitors have contradictory effects on its brain disposition and analgesic effects [3,33]. P-gp inhibition did not alter the apical-basolateral (A-B) absorption of oxycodone, but let to reduced basolateral-apical (B-A) secretion, suggesting that only the P-gp located at the apical layer of Caco-2 cells could affect oxycodone transport. In P-gp KO mice, brain levels of oxycodone were increased compared to wild-type animals. Oxycodone may also upregulate P-gp in a dose-dependent manner, as shown by a higher rate of ATP consumption. Administration of oxycodone induces P-gp approximately by 2-fold, 4-fold, 1.6-fold, and 1.3-fold in the intestine, liver, kidney, and brain, respectively, thus resulting in lower absorption and increased elimination of oxycodone itself, with possible tolerance development. Oxycodone-induced P-gp upregulation may be the source of chemotherapy resistance, since its co-administration with paclitaxel, a P-gp substrate, was associated with reduced concentrations of this chemotherapeutic drug by 90% in the liver, 87% in the kidney, 38% in the brain, and 70% in plasma [35].

The impact of P-gp on the pharmacokinetics of fentanyl is still unclear since some studies have not identified this strong opioid as a P-gp substrate [3], and data about its P-gp-mediated transport are contradictory. While some studies did not find a correlation between P-gp levels and fentanyl transport [4,7], others showed that P-gp inhibition increased the passage of fentanyl through the BBB towards the CNS, with consequent enhancement of its antinociceptive and side effects (e.g., dose-dependent respiratory depression and prolonged duration of the loss of righting reflex). Similar findings have been shown in P-gp KO animals. P-gp inhibition also leads to increased oral fentanyl absorption and plasma concentrations [92]. While in vitro studies suggest fentanyl to be a P-gp inhibitor [83], it has been found to activate P-gp ATPase activity [92].

Fentanyl derivatives such as alfentanil and sufentanil do not act like P-gp substrates in vitro [3]. However, alfentanil was proved to be a P-gp substrate in P-gp KO mice [93]. Similarly to fentanyl, these derivatives inhibited P-gp in Caco-2 cells [83]. To the best of our knowledge, there are no data suggesting sufentanil may act as a P-gp substrate in vivo. Sublingual sufentanil, indeed, displays fast and repetitive onset of action and rapid equilibrium between plasma and CNS concentrations, providing clinical evidence for the rationale behind its use in post-operative patient-controlled analgesia (PCA) [94,95]. The rate of equilibrium between the plasma and the effector site is a key parameter to be considered in the choice of the optimal opioid for PCA, because a delayed equilibration may lead to an initial overshoot of drug administration, which significantly increases the risk of opioid-related side effects [96].

Hydrocodone seems to act as a P-gp substrate, as shown in P-gp deficient mice; however, this finding was not confirmed in bidirectional transport assays on MDR1 and Mdr1a-MDCK transfected cells. Hydrocodone does not inhibit human P-gp mediated efflux of calcein-AM even at high concentrations (>100 μM) [7].

Tramadol and O-desmethyl-tramadol do not act as P-gp substrates in Caco-2 cells [97,98].

Buprenorphine does not act as a P-gp substrate in a bidirectional transport assay with either human MDCKII-MDR1 or Caco-2 cells, nor in P-gp deficient mice [7]. Nonetheless, P-gp inhibition led to increased brain uptake of buprenorphine and respiratory depression in animals [99]. Injection of cyclosporine A, quinidine and verapamil, increased brain uptake of [^3^H] buprenorphine by 1.5-fold, while vinblastine and vincristine at 0.1 mM did not have this effect; elimination of [^3^H] buprenorphine from the brain was inhibited by 32–64% after administration of cyclosporin A, quinidine, verapamil, or vinblastine. Buprenorphine has a molecular weight of 467.6 and is a highly lipophilic, organic cationic drug, positively charged at physiological pH; therefore, it may potentially act as a P-gp substrate [100]. On the other hand, norbuprenorphine was found to be an avid P-gp substrate both in vitro [7] and in vivo [101]. Whether tapentadol is a P-gp substrate is still unknown.

Pentazocine (PTZ), an opioid agonist/antagonist, was proved to be a P-gp substrate in vivo [102].

Loperamide is a potent opioid receptor agonist, but shows no remarkable CNS effects, because of its poor absorption in both the gastroenteric tract and the brain. In fact, P-gp hinders the entrance of loperamide in the CNS and the intestine with an efflux/influx ratio of 10. For this reason, loperamide is used in clinical practice only as an antidiarrheal agent. Administration of P-gp inhibitors leads to increased transport of loperamide in the CNS and caused opioid-related side effects (respiratory depression). On the other hand, tariquidar-induced P-gp inhibition does not alter loperamide effects on the CNS when administrated at 2 mg/Kg: this dose was probably too low to inhibit P-gp at the BBB, while 8 mg/Kg tariquidar generated a variation in brain uptake of 11C-loperamide as its radioactivity was increased by 3-fold [7,103]. Coadministration with spironolactone resulted in antinociceptive effects in rats, suggesting that P-gp inhibition may allow loperamide to pass across the BBB and act centrally [104]. P-gp knockout mice had a 13-fold higher accumulation of loperamide in the brain [33].

Meperidine is believed to be a P-gp substrate in vitro but not in vivo: P-gp knockout mice had no greater antinociception [33].

Methadone is approved for opioid-use disorder (OUD) maintenance therapy. Specific ABCB1 polymorphisms are associated with variability in serum methadone concentration over the 24 h dosing interval for subjects on methadone maintenance therapy (MMT) [105]. In fact, methadone was proved to be a P-gp substrate, with an efflux ratio of 2.61. When a P-gp inhibitor and methadone were coadministrated in P-gp KO mice, opioid-induced analgesia improved because its brain concentrations increased [33]. Accordingly, methadone-induced antinociception in mice was reduced after induction of P-gp in brain capillaries [20]. Despite its stereoselectivity for methadone enantiomers being slight, P-gp transports the (+) S-enantiomer 10% more than the (R)-enantiomer [33], the latter being accountable for methadone MOR agonism at the CNS [4]. Furthermore, P-gp interferes with the intestinal absorption of orally administrated methadone: this may account for the inter-individual variability in methadone-induced clinical effects. On the other hand, quinidine-induced P-gp inhibition did not affect the PK of intravenous methadone [7]. Methadone also inhibits P-gp activity in vitro [7,45].

P-gp-mediated transport of nalbuphine is inhibited by elacridar, so it is believed to be a P-gp substrate at the BBB. This agonist–antagonist, however, does not inhibit P-gp [7].

Diprenorphine, a non-selective opioid receptor antagonist, is believed not to be a P-gp substrate since elacridar and verapamil do not alter its transport in Caco-2 cells. Diprenorphine seems to be transported by P-gp in MDCKII-MDR1 cells. These results were not confirmed in mice and there are no data on humans [7].

MOR antagonists naloxone and naltrexone do not act as P-gp substrates [106,107]; naloxone was found to weakly inhibit P-gp, only at high doses (>100 μM) and with very low affinity [107]. However, data about naloxone are contradictory. Naltrexone does not inhibit P-gp [7].

### 2.6. The Role of P-gp in Drug–Drug Interactions

In the last few years, most of the literature concerning drug–drug interactions (DDI) has focused on the key role of the cytochrome P450 system. The risk of DDI when treating chronic pain in patients with comorbidities has been mainly attributed to drugs acting on the metabolizing enzymes CYP2D6 and CYP3A4, in terms of genetic variability, metabolizer status, effects of inducers and inhibitors, and the risk of interaction in polypharmacy [108]. In chronic pain patients with comorbidities, opioids without or with a minor impact on the cytochrome P450, such as morphine, hydromorphone, and tapentadol, can be considered a first choice of treatment [109,110]. The role of P-gp in drug–drug interactions is an emerging topic in clinical practice.

Many drugs acting as substrates for P-gp are also substrates of CYP3A4. However, there are some drugs, such as dabigatran, which are exclusively substrates of P-gp and for which the severity of the interaction on the clinical level is unclear. In a recent study by Akamine et al., 2019 [111], authors showed on the basis of the available data that the exclusive inhibition of P-gp, for those drugs that are not substrates of CYP450, or the P-gp inhibition in the absence of a CYP450 inhibition, have modest effects on the pharmacokinetics of the substrate drug and very little clinical relevance. Conversely, the induction of P-gp by rifampicin and some anticonvulsants (including carbamazepine and phenytoin) has significant clinical effects on drugs which are exclusively substrates of P-gp. Therefore, extrapolating, one must pay attention mainly to the phenomena of induction rather than inhibition of P-gp in case of potential DDI. However, the clinical consequences of P-gp inhibition also depend on the severity of the pathologies affecting the liver, kidney, and heart of the specific patient.

Another relevant issue is whether the P-gp expressed in the intestine, liver, kidney, and BBB are identical, have the same affinity for the same substrate, and are equally affected by the interactions with the same inhibitor, normalizing for the different concentrations of inhibitor that occur in the different districts, or if there are differences. In a study based on the use of KO mice for P-gp, the administration of risperidone caused identical plasma concentrations compared to wild-type mice, suggesting the lack of any role for intestinal, hepatic, and renal P-gp. By contrast, the brain concentration of risperidone and its metabolite was 10-times higher in KO mice than wild-type mice, thus suggesting that risperidone is a substrate for the brain P-gp only [112]. Therefore, transporter-mediated DDIs at the BBB may occur in some cases without changes in drug plasma pharmacokinetics, so that drug concentrations in the brain need to be considered in order to assess such DDIs [113].

### 2.7. Expression of P-gp during Aging

In PET human studies, it is known that the non-marketed third-generation ABCB1 inhibitor tariquidar, which inhibits ABCB1 at the human BBB, is able to cause up to fourfold increases in (R)-[11C]verapamil brain uptake [114]. The same experiment carried out in young and elderly people showed that the passage of verapamil, thus the intensity of P-gp inhibition, was significantly higher in elderly than in young subjects, because of a reduced expression of P-gp in the former [115]. Therefore, greater attention must be paid in the elderly patients.

### 2.8. P-gp and Peripherally Acting Mu-Opioid Receptors Antagonists (PAMORAs)

Opioid-induced bowel dysfunction (OIBD) is one the most frequent adverse effects when engaging in opioid prescription for both chronic cancer and non-cancer pain (CNCP): it includes various signs and symptoms, namely nausea, vomiting, xerostomia, gastro-esophageal reflux, and constipation. Unlike many other opioid-related side effects, opioid-induced constipation (OIC) is particularly bothersome since tolerance is rarely reached, potentially leading to opioid discontinuation and consequent poor pain control [116,117]. Since OIBD is mainly caused by opioid agonism towards μ receptors (MOR) located throughout the whole gastrointestinal tract, peripherally acting mu-opioid receptors antagonists (PAMORAs) have been developed in order to avoid or revert these troublesome effects while sparing central analgesia. Currently available PAMORAs include naldemedine, methylnaltrexone, and naloxegol. Naldemedine is an amide derivative of naltrexone, added with a side chain (2-(3-phenyl-1,2,4-oxadiazol-5-yl)propan-2-yl)acetamide increasing both its polar surface and molecular weight, thus hindering its passage through the BBB [118]. Naldemedine is indeed a P-gp substrate, since its Cmax and AUC were increased when coadministered with cyclosporine, most likely due to inhibition of intestinal P-gp and consequent enhanced bioavailability of naldemedine; higher frequency of naldemedine-induced adverse effects confirmed these findings, although the administrated dosage of naldemedine was higher (0.4 mg) than the recommended dose [119]. In fact, clinical trials enrolling subjects with both cancer and non-cancer pain showed naldemedine is effective and well tolerated when administrated at a 0.2 mg once daily dosage [118]. On the other hand, the role of P-gp in expelling naldemedine out of the CNS seems to be only marginal, since mdr1a/b KO mice showed low naldemedine brain-to-plasma concentration ratio (brain Kp < 0.1), even if brain Kp itself was relatively higher (4-fold) when compared to wild-type mice [120]. Naldemedine does not inhibit P-gp [121]. Hence, its use may minimize the risk of drug–drug interactions (DDIs) when administrated alongside other P-gp substrates. Moreover, naldemedine could prevent or reduce opioid-induced nausea and vomiting (OINV), given its ability to act in certain brain regions, namely the area postrema, that are not protected by the BBB [120].

Methylnaltrexone is not a P-gp or a CYP3A4 substrate [116]. Therefore, its activity is confined outside the CNS, because of its chemical structure, where a quaternary ammonium has been added to naltrexone to limit its ability to cross the BBB. When subcutaneously or orally administrated, methylnaltrexone does not induce opioid withdrawal or interfere with opioid analgesia [122].

Conversely, naloxegol, which is a PEGylated derivative of naloxone, is a P-gp substrate: hence, its penetration in the CNS is scarce, allowing it to reduce OIC while preserving central analgesia [123]. Transport by intestinal P-gp is saturable at high naloxegol concentrations. Caution must be observed when administrating naloxegol alongside P-gp and CYP3A4 inducers and inhibitors, since the possibility of DDIs is relevant [124].

### 2.9. P-gp and Neuropathic Pain Medications

Neuropathic pain is defined as “pain caused by a lesion or disease of the somatosensory nervous system” and is characterized by typical features, such as hyperalgesia and allodynia. Many painful conditions may be associated with neuropathic components, so that 7–10% of the general population is believed to suffer from neuropathic pain [125]. Structural changes and permeability of the blood–spinal cord barrier (BSCB) [126], the blood nerve barrier [127], and the BBB [128] may be crucial factors for development of neuropathy in several pathological states, ranging from diabetic neuropathy [129], trigeminal neuralgia [130], radiculopathies [131], and migraine [66,132]. Particularly, abnormal expression of BBB proteins, namely laminin and ZO-1, has been described during cortical spreading depression (CSD), the latter typically occurring during migraine attacks [66]. Several antimigraine drugs were found to be P-gp substrates [9,133]: for instance, P-gp may be in an “intermediate” conformation when binding to both sumatriptan (STT), the first triptan drug to ever be developed, and eletriptan (ETT), a second generation triptan drug. Nonetheless, STT acts as a weak P-gp substrate (efflux ratios from 1.1 to 2.9) and inhibitor, with a <10% reduction in ATP hydrolysis: this phenomenon is probably due to a “conformational barrier” to ATP hydrolysis. Conversely, ETT is a stronger P-gp substrate (efflux ratios from 11 to 46.7), and is associated with a 2-fold enhancement in P-gp activity, with no more structural impediments to ATP hydrolysis [9]. As one would anticipate, several double-blinded placebo-controlled studies have assessed the superiority of ETT to STT in terms of relief of headache and associated symptoms (e.g., nausea, photophobia/phonophobia), improvement of functioning and consequent reduction in use of rescue medications, and so on. Nonetheless, these head-to-head comparisons enrolled cephalalgic patients with no other clinically relevant comorbidities [134,135,136,137,138], thus likely excluding the possibility of drug–drug interactions: this kind of scenario is unfortunately quite rare in clinical practice. Hence, assessment of mere relative superiority of one drug to another could turn out to be limiting when choosing appropriate therapies, while a “patient-based” approach is preferred. The use of non-pharmacological options for neuropathic pain, namely transcranial magnetic stimulation (TMS) and transcranial direct current stimulation (tDCS), could rely on their impact on nervous system barriers.

Subjects with both cancer and non-cancer pain may present with a neuropathic component (so called “mixed pain”). For instance, almost 80% of patients suffering from non-malignant back pain experience neuropathic signs and symptoms [139]. Neuropathic features are also commonly reported in cancer patients, and they can either be adverse effects of chemotherapeutic drugs (chemotherapy-induced neuropathic pain, CINP) or be the indirect consequence to immunosuppression. According to guidelines, first-line treatment of neuropathic pain includes antidepressants, such as tricyclic antidepressants (TCAs), venlafaxine and duloxetine, and antiepileptic drugs, namely gabapentin and pregabalin [140]. In particular, pregabalin binds to voltage-gated calcium channels alpha2-delta (α2δ) subunits and is indicated in epilepsy, diabetic neuropathy, fibromyalgia (FM), and trigeminal neuralgia [141]; moreover, recent findings suggest its potential benefit for postoperative pain management [142]. However, recent findings hint that pregabalin may act as a P-gp substrate in mice, since pretreatment with P-gp inhibitors prolonged the anti-hyperalgesic effects of intraperitoneally (i.p.)-administrated pregabalin from 3 h to 72 h in an intermittent cold stress (ICS)-induced FM-like pain animal model. Still, studies assessing whether P-gp is directly responsible for efflux of pregabalin out of the CNS are needed [141]. Efficacy and tolerability of opioid analgesics in treating neuropathic pain are still under open debate [140]. “Dual” opioids may help control neuropathic features, as is the case for tramadol [143] and tapentadol. The latter was found to be beneficial for patients with back and neck pain presenting with neuropathic components [139]: particularly, tapentadol was superior to oxycodone/naloxone with regard to neuropathic features control, and in terms of tolerability, since classical opioid-induced adverse effects, namely constipation (OIC), nausea and vomiting (OINV), were much less frequent [144]. Similarly to tramadol, tapentadol has a dual mechanism of action: still, given its negligible effect on the serotonin transporter (SERT), only norepinephrine reuptake is inhibited, giving tapentadol a better tolerability profile [145]. Besides, serotonin is known to have pronociceptive qualities and is commonly released in injured sites in the case of acute pain, thus predisposing a patient to pain sensitization [146,147]. Hence, tapentadol may have an important role in reducing incidence of pain sensitization and chronicization, compared to other opioids. Furthermore, affinity of tapentadol to MOR (“μ-load”) is 50-times lower than morphine, thus reducing the rate of classical opioid-related adverse effects [145]: this is particularly important for subjects dealing with cancer pain, since they often experience constipation, nausea, vomiting and a lowered quality of life generally, not only due to opioid use, but also because of chemotherapy-induced side effects [148].

### 2.10. P-gp and Non-Opioidergic Pain-Relievers

Besides opioids, ABC transporters, including P-gp, on brain microvascular endothelial cells, may be modulated by other drugs that are commonly used for pain management, namely dexamethasone and acetaminophen (APAP; i.e., N-acetyl-p-aminophenol, paracetamol). These compounds are able to activate nuclear receptors, such as the above-mentioned constitutive androstane receptor (CAR) and pregnane-X-receptor (PXR), thus resulting in P-gp overexpression and consequent reduction in blood-to-brain transport of opioidergic drugs that act as P-gp substrates [149]. This may account for the effectiveness of certain fixed combinations between opioid and non-opioid drugs, such as codeine, hydrocodone, and oxycodone, which are available as immediate release formulations in combination with acetaminophen. According to the dosage of opioids, these fixed combinations can be used for mild to moderate (codeine/acetaminophen) [91] and for moderate to severe (hydrocodone/acetaminophen and oxycodone/acetaminophen) [150,151] chronic pain management. Since these opioids are not P-gp substrates, acetaminophen-induced P-gp upregulation should not have detrimental effects on opioid-induced analgesia when these drugs are combined in fixed formulations.

## 3. Conclusions

The importance of P-glycoprotein as the main obstacle to drug delivery to the brain and CNS has been widely demonstrated, particularly by using knockout animals. Despite, in physiological conditions, its role being essential for preventing toxins and other potentially harmful agents to cross the BBB, in pathological conditions its activity may represent a severe limiting factor to adequate analgesia. This efflux protein is, indeed, the main molecular cause for pre-clinical and clinical drug failure. However, probably in clinical practice, this concern is still widely underestimated. Physicians should be aware of the possible negative consequences of using polypharmacy, including molecules with different activities on P-gp in cancer pain patients. Limiting the efficacy of chemotherapy or impairing the analgesic activity of opioid analgesics may significantly affect expectancy and quality of life of chronic pain patients suffering from cancer.

Most opioids are known to be P-gp substrates; therefore, increased expression of P-gp potentiates their efflux and prevents their delivery in the central nervous system (right side). Opioids may also modulate P-gp activity: morphine and oxycodone are P-gp inducers, while buprenorphine and methadone are inhibitors of ATP-Binding Cassette (ABC) transporters at the blood–brain barrier (BBB). Other drugs commonly used in pain management may modulate P-gp expression at the BBB, through intracellular mechanisms of nuclear transcription. Dexamethasone and paracetamol bind the nuclear receptor (NR), which, after dephosphorylation by a protein phosphatase (PP), translocates to the nucleus and binds its response element on a target gene, leading to protein transcription and overexpression of ABC transporters, such as P-glycoprotein (P-gp), on the membrane of brain microvascular endothelial cells (left side).

## Data Availability

Not applicable.
